# Filling Cervical Cavities

**Published:** 1895-02

**Authors:** 


ARTICLE II.

### FILLING CERVICAL CAVITIES.

The form of the cavity for retention of the filling, and ample extension of the cavity for the prevention of recurrence of decay, are the two main items in this class of cavities to secure duration of fillings. Other factors belong to the method, as contour and contact, triangular form of the interproximate space, secured usually by wedging or pressure; and the adaptation of the filling material to the restoration of form. Let us particularize a little. To prepare a cavity, always cut till you come to something good at the cervical margin, even if it should be necessary to cut all the enamel away at the cervix. Cut wide buccolingually, extending to the angles of the cusps and including them if thin or of poor texture.

The cusp angles are not of much importance in supporting the filling by this method of anchorage, and should be cut away till the strongest possible border can be secured, and restoration be made with the filling.

I have observed more failures (recurrences) at this point (cusp angles) than at any other point.

This is due largely, as I think, to the former practice of leaving these angles intact, with the view of giving some support to the filling, which I believe to be a fallacy. When but little dentine is left it is entirely inadequate to sustain the easily cleaved enamel under the wear and tear of mastication.

Anchorage gives better access to the cavity margins and all parts of the cavity likewise, than the older forms.

'Outward bevel all round the cavity margin (not too great) and the cavity is ready for the filling.

We go to our meetings, and as we meet elsewhere, we talk and talk about how to prepare cavities and introduce fillings to make them last. Now what more is there to it than the putting of all the material you can into a good clean hole?

However simple the general principles may seem, experience teaches many that there is a wide difference as to who cleans the hole and puts in the material—whether the results are to be permanent or temporary; comfortable or painful, heaven-like or its antithesis—whether by the master or by the apprentice, the accomplished operator or the bungler.

The cervical margin! Place your filling material evenly and firmly on all margins and surfaces alike, maintain as uniform destiny as possible through the whole mass of the filling. Restore the lost form of the tooth caused by decay and excavation, finish, collect fee, benediction—the cervical margin should now be able to take care of itself.

Let me observe that the signs of the times indicate that amalgam is to take rank as a permanent filling material, both for rich and poor, and as I think, on its merits in resisting decay, easier form-building in general, and particularly for its adaptability to the cervical margin.—*Review.*

